# Does living arrangement matter? Analyzing relations from Chinese women's perspective with cultural change

**DOI:** 10.3389/fsoc.2025.1516890

**Published:** 2025-04-09

**Authors:** Lu Ma, Jia Xu

**Affiliations:** ^1^School of Law, Anhui Normal University, Wuhu, China; ^2^School of Marxism, Anhui Normal University, Wuhu, China

**Keywords:** living arrangement, women, family relations, rural, cultural change, China

## Abstract

**Introduction:**

Due to the decrease in family size and lower fertility rate in China, traditional patrilineal-centered living arrangements have become ineffective, prompting families to change them to meet their immediate and long-term care needs for families. This study examines the extent to which Chinese rural women's living arrangements improve their family relationships after marriage.

**Methods:**

This study utilizes institutional regulatory documents at the national level and observational data from 109 semi-structured interviews with married women (aged 22-89 years), which were conducted in 2019 and 2023. Using data from interviews with married women, we examine the family structure, family expectations, and family care contribution to analyze women's different living arrangements in rural areas of southern China.

**Results:**

It demonstrates that women's different living arrangements have varying effect on family relationships. First, the family structure, suggesting that married women's relationship with their natal family after marriage has great impact on women's living arrangement after marriage. Second, the possibility of children inheriting the family name enhances family relations, consistent with family expectations, as the family name identifies the family members. Third, family relationships may be strengthened through providing frequent and long-term care to their natal family for married women.

**Discussion:**

We find that the women's post-marriage living arrangements help strengthen their family relationships in a balanced manner and address unmet family care needs under the strong influence of Confucian cultural ideals. Our findings suggest that changes in living arrangements explain family relations and gender equality within the Chinese context of an aging society.

## 1 Introduction

This study aims to explore how rural families enhance their family relations by practicing different living arrangements as a possible solution for strengthening family care obligations and the changing cultural values of Confucianism in family relations in rural China. Married women rely on family support to care for children and older parents. However, when they have to work to increase household income, traditional patrilineal-centered living arrangements become ineffective in addressing the balance of work responsibilities and family care. This has prompted families to change their living arrangements to meet their new needs. Further, changes in the living arrangement is not smooth, the progress is full of negotiation and tugging, and even led to family conflict, such as in cases where the dual living arrangement has changed the power structure of the family. Older people, who are strongly influenced by Confucianism-based traditional family ethics, believe that this living arrangement undermines the power of the husband's family, restricting their strict control over their daughter-in-law and destroy the family relations (Wong, 2016).

Further, it is necessary to consider a systematic analysis of the extent of family care contribution in the association between living arrangement changes and family relations. Such an investigation is particularly necessary in rural China since older people's income in these regions is relatively lower than incomes of those in urban areas; thus, family support for balance care needs and living arrangement might affect the quality of life and well-being of rural older adults. It is common for the average pension in rural areas to be inadequate to support older people. Some rural pensioners in central and eastern China receive an annual pension of RMB 1,200–1,800 (i.e., US $180–270), in addition to medical care insurance (Chen, 2016; Du and Liu, 2014).

Therefore, this study argues that different living arrangements after women's marriage led to changes in their family relations. The decision about how to arrange their lives and how to get along with their relatives or families after marriage has become critical for women. However, it is not well understood under Chinese cultural ideals, particularly from the women's perspective.

In addition, this study argues that the changes in Chinese family living arrangements contribute to gender equality. The traditional Confucianism argument is alive and well in its understanding of filial piety, traditional gender roles, and familial ideology in Asian countries (Sung and Pascall, 2014; Phillips and Jung, 2013). Under Confucian values, gender issues have been discussed as a common concept in balancing family members' obligations in work–care relationships in society (Phillips and Jung, 2013; Yeh et al., 2013; Chen, 2016). However, the Chinese family's role in supporting gender development is different (Yeh et al., 2013; Chen, 2016). Gender equality differs from the standard academic concept worldwide of, which looks at emphasizing the leading and core role of harmony between family members in society (Yeh et al., 2013; Zhan, 2004). Gender harmony is heavily reliant on filial piety and the responsibilities and obligations related to intergenerational family care. The practice of the traditional Chinese family exemplifies the gender harmony ideology through a division of labor based on gender roles. Men are typically responsible for external work and financial support, while women usually assume domestic responsibilities such as housework and caregiving. Family members with different gender take relevant gender role while taking the relevant responsibility according to filial piety value. Younger female generation priorities their care obligations toward their parents against other responsibilities, while older generation accepting it also based the value of filial piety, by which caring for older family members should be motivated by love and respect toward their parents rather than any forms of rewards such as monetary feedback (Feng, 2017). It should be noticed that this gender harmony is different from gender equity. Actually, gender harmony does not include the idea of equity or inequity, instead the traditional Confucian values change gender equity to the idea of gender harmony to avoid conflicts related to gender-based problems in society. So that younger generation take their family care responsibility due to value obligation and family task differences instead of thinking whether these family obligations are equal or not. Therefore, we argue that it is also important to examine how far living arrangements impact the structuring of family relationships while also influencing gender harmony under Confucianism in rural China.

This study utilizes institutional regulatory documents at the national level and observational data from 109 semi-structured interviews with married women (aged 22–89 years), which were conducted in 2019 and 2023. It demonstrates that women's different living arrangements have varying effects on family relationships. The first assumption concerns the family structure, suggesting that married women's relationship with their natal family after marriage has great impact on women's living arrangement after marriage. Second, the possibility of children inheriting the family name enhances family relations, consistent with family expectations, as the family name identifies the family members. Third, family relationships may be strengthened through providing frequent and long-term care (e.g., for over 10 years) to their natal family for married women.

## 2 Conceptual framework and literature review

### 2.1 Chinese living arrangement and relevant traditional value

Along with intensive labor market participation, adult women, whether as wives, daughters, or young mothers, strive to balance their work and family lives. However, they also grapple with the question of who will care for older people and children. With the rapid rise in the aging population and weak public care services, meeting the care needs of family members can be challenging (Wang and Zhan, 2021). Additionally, rural areas in China lack a comprehensive public healthcare system for those in need of care. Rural community-based care serves as a secondary care service, with institutional care as a backup. The reform of the Chinese care system shifts the responsibility of caring for children and older people back to the family, aligning with the core traditional value that the family should provide primary care.

This traditional value contributes to the reason public welfare spending on individuals remained low in China for a long time. Family members are expected to support each other under cultural norms (Chen, 2016) such as strong family-based care provisions, loyalty, filial piety, and a strict division of gender roles (Kim, 2015). Accordingly, parents and children have a mutual obligation in which parents must provide care and raise children, while adult children must provide care for older parents, based on filial piety which rooted in the cultural value in Confucianism (Du, 2013; Feng, 2017; Peng, 2014; Peng and Chien, 2018). Based on this value point, younger generation should obey and respect the older generation unconditionally, and legal responsibility to provide care for their parents and to offer emotional and financial support toward their parents (Peng and Chien, 2018). Furthermore, Confucianism, which emphasizes loyalty, obedience, and hierarchy, has extended these cultural ideals to successive generations of family members. Children in the next generation also have obligations to care for their grandparents in old age. In return, grandparents provide family support to raise their grandchildren when young parents are working or unable to take care of their children for various reasons. This is referred to as a “family feedback system” (Chau and Yu, 2013; Fei, 2006; Peng, 2014), and it partially explains why individual care needs are traditionally considered a family matter rather than a societal or state responsibility (Sung and Pascall, 2014).

The natal family and daughters' role in Confucianism-based families presents a dilemma between caring for one's in-laws and caring for one's own parents, which for women is significantly different after getting married. On the one hand, in Chinese culture, the natal family refers to the family into which a woman was born, including her parents, siblings, and brothers' wives and children. Moreover, the husband's family includes his parents and his brothers' families (Judd, 1989). Married women do not have the obligation to support their natal parents and do not have the right to inherit their natal parents' property. This is because they are considered members of their husbands' families and become outsiders to their natal family after marriage (Wong, 2016; Lei, 2013). In the Chinese context, the expression “split water” is used to describe married women. On the other hand, considering filial piety in patrilineal living arrangements within the framework of Confucianism-based family ethics, a married woman is expected to fulfill her obligations and demonstrate greater filial piety toward her husband's family, especially toward the parents-in-law, rather than her own parents (Yang, 1996; Zhang, 2007). Owing to her husband's strict obligation to support his parents, his wife not only shares the same responsibility with her husband but also takes on more family care duties than her husband. This is because her role in her husband's family as a daughter-in-law involves responsibilities such as helping with childbirth, caring for the family, and supporting parents in old age (Mao and Chi, 2011; Whyte and Xu, 2003).

Nevertheless, for married women, family relationships with their natal family members are typically important, although their role in the natal family may change (Judd, 1989). They continue to visit their natal family, especially during the first few years of marriage, until their parents die (Wong, 2016). Although daughters do not have a duty to support their parents nor have inheritance rights, they share many emotional interactions. It is argued that even short visits to their natal families are a relaxing and joyful experience for married women, and a way for them to express their sense of filial piety (Xie and Zhu, 2009). If a married woman never or seldom visits her natal family for any reason, she might be criticized as unfilial (Xie and Zhu, 2009). Similarly, the parents of married women also visit their daughters and even prepare gifts for her and her husband's family during holidays in Chinese society. Indeed, we found that in the last 20 years, the relationship between married women and their natal families has become closer than ever (Zheng and Di, 2018), with more married women becoming as important as sons in terms of maintenance obligations (Cong and Silverstein, 2008). Under this background, some married women tend to change their living arrangements to enhance their relationship with their natal family and look for care support from their natal family (Li, 2024).

Consequently, it is significant for women after marriage to arrange their living arrangements to balance families care needs. Literature proposes different types of living arrangements in Chinese society for women after marriage, including (i) patrilineal-centered living arrangements, in which a woman lives with her husband and parents-in-law after marriage; (ii) matrilineal-centered living arrangement, in which a woman and her husband live with her natal family members after marriage; (iii) dual living arrangement, under which the woman and her husband alternately reside with each set of parents for part of the year after marriage; and (iv) residing without any parents, in which the woman and her husband move into their own home after marriage (Zhang and Zhai, 2013; Wu and Peng, 2017; Huang, 2013). By choosing different family living arrangements, it is argued that family relations can be strengthened due to increased frequency of interaction between family members when they live in the same household (Roberts et al., 1991) or weakened due to decreased mutual support from generations since they live independently as a nuclear family (Lüscher, 2002). Family relations could become tense since married women might have to learn to deal with relationships with their parents-in-law, such as in a patrilineal-centered living arrangement (Zhang, 2009).

### 2.2 The patrilineal-centered living arrangement

In traditional Chinese society, family relationships have a long history around patrilineal bloodlines that form the basis of the patrilineal-centered living arrangement (Fei, 2010). Women leave their natal families and marry into their husbands' families for a patrilocal marriage (Huang, 2013). In patrilineal-centered living arrangements, it is common for a woman to live with her husband and parents-in-law after marriage. Typically, the wife and husband have a separate room in the apartment or home, which the husband's parents own. The woman fulfills the task of continuing the husband's genetic line and assumes the obligation of supporting the husband's parents in old age. Similarly, the husband's family provides shelter for young couples and endows the husband and their sons with the right to inherit their property (Fei, 2006). In patrilineal-centered living arrangements, married women (as wives) are subordinate to their husbands. The wives cannot make decisions, enjoy the benefits of family relations, or contribute to strengthening or weakening family relations (Shiga, 2003). Meanwhile, adult male members (their brothers and husbands) hold the core position in the family and are fully responsible for strengthening the relationships between family members and generations (He, 2019). The family is divided when the adult sons marry and start new careers. The sons may choose to divide the land and property equally from their natal family with their brothers (Fei, 2010). The literature illustrates that traditional family relations in rural China are influenced by the male perspective, which is highly correlated with the prevalence of patrilineal-centered living arrangements (Cong and Silverstein, 2012).

### 2.3 The matrilineal-centered living arrangement

The matrilineal-centered living arrangement involves a woman with no brothers and her husband living with her natal family members after marriage. In this arrangement, the husband lives with his wife's parents, supports them, and allows his children to take on the wife's surname after marriage (Fei, 2006, 2010). A woman can inherit from her parents, but the husband is ineligible from inheriting his birth parents' property (Li, 2003; Du, 2021). Such matrilineal-centered living arrangements are often considered abnormal because they challenge traditional male-dominated cultural values, especially in rural China. This type of living arrangement is only accepted by the husband's family if they are very poor and do not have the ability to provide financial support to the newly married couple (Fei, 2006). In Chinese society, the husband in such a marriage is labeled Shangmen Nvxv (上门女婿), which implies that he severs ties with his birth family in terms of rights and obligations. He does not inherit his parents' property or provide them with old-age care (Li, 2003; Du, 2021). This means that he is too weak to maintain his patrilineal bloodlines. The aims of such marriage formations are for the wife to assume the role of a son, thereby strengthening her family relationship with her natal family, in order to increase the resources for caring for older family members and continuing the family's genetic line.

### 2.4 The dual living arrangement

From the woman's perspective, family relations have continued to change in contemporary Chinese society. On the one hand, women are “kin-keepers,” contributing to family care and supporting the family's financial income between generations, which has become more important than ever (Xu, 2021). On the other hand, the implementation of the one-child policy and the development of the social economy have reduced the fertility rate and strengthened parent-child relationships in China. This has been reflected in the dual living arrangement under which the woman and her husband alternately reside with each set of parents for part of the year after marriage. The families of the woman and her husband are expected to prepare a new shelter and provide furniture for the new couple. Comparing the data from the Chinese General Social Survey (CGSS) in 2013 (Wu and Peng, 2017) and the 2005 National Population Sample Survey (Zhang and Zhai, 2013), we find that the patrilineal-centered living arrangement in rural China remains the dominant living arrangement for young couples (Zhang and Zhai, 2013; Wu and Peng, 2017). However, data also show that dual living arrangements have been largely accepted and adopted in society (Wu and Peng, 2017).

Usually, women and their partners come from the same place, either the same village or the same county. They meet and develop their relationship when they are studying or are introduced by relatives and friends who live nearby (Liang and Dai, 2022). Therefore, most women's partners originally live in nearby as the women's families. Consequently, a woman's family will do their best to provide relatively good living conditions to attract the woman's partner to be willing to live with the woman's family. Under such circumstances, both the woman and her partner are responsible for the care of their parents, and both can inherit from both sets of parents (Shiga, 2003; Li, 2003; Du, 2021). The dual and matrilineal-centered living arrangements share similarities and differences. For example, married women prefer to spend as much time as possible with their natal family in both; however, new couples avoid social stigma by choosing to split their time between both families and naming their children by their husband's family name (Du and He, 2017; He, 2019). Nevertheless, under dual living arrangements, the husband can still inherit from his family, a practice not possible in matrilineal-centered living arrangements (Gruijters and Ermisch, 2019).

### 2.5 Residing without any parents

The rapid development of China's economy has enabled young farmers to leave the countryside and live in cities and towns to reside without any parents (Gruijters and Ermisch, 2019; Wang, 2019); that is, the woman and her husband move into their own home after marriage and, in principle, share the housework and family care responsibilities. However, the woman is ineligible to inherit from her birth parents, while her husband can inherit from his birth parents (Gruijters and Ermisch, 2019). In rural areas, women who live independently, either to study, work, or through marriage, are seen as affluent or elite. Additionally, it is common for these couples to live far from their parents (Gruijters and Ermisch, 2019).

## 3 Analytical framework

### 3.1 Dimensions to measure living arrangements and family relationships

The living arrangements discussed in this paper refer to how married women choose to live in the household to balance families' care needs and work as rural areas, such as living with natal parents or with parents-in-law in the same household or both, or living within a nuclear family. Women's choices might have different impacts on their family relationships depending on the organization of the family structure, family members' expectations, and women's ability to provide care (Guo et al., 2020; Hank, 2007; Li et al., 2021).

For married women, different members and combinations directly affect the family relationship and inheritance eligibility. Forming living arrangements requires family members to form a stable family structure to maintain intergenerational succession and convention (Gruijters and Ermisch, 2019). As such, this study takes the “family structure” as its first dimension to analyze the interrelationship between living arrangements and family relations.

To measure how much family structure contributes to the association between living arrangements and family relations, we include the following measurement criteria: (1) whether the married woman has a brother and (2) the distance between the residences of the married woman and her natal family after marriage (see Table 1). Based on our analytical approach, we assume a strong level of family relationships when a woman does not have brothers and continues to live in the same village as her natal family after marriage. An intermediate level of family relations is evident if a woman has brothers and continues to live in the same village as her natal family after marriage. Weak family relations are identified when a woman has brothers and does not live in the same village as her natal family after marriage.

**Table 1:** Relationship between women's living arrangements and family relationships.

	**Family relations**				
**Living arrangement**	**Family structure**		**Family expectations**		**Ability to provide care**
	Whether the married woman has a brother	Distance between the residences of the married woman and her natal family after marriage	Perspectives of the woman's birth parents of their married daughter's role in the family	Surname of the children born to the woman	Association between living arrangements and family relations
PC^a^	Weak to medium		Weak		Weak
MC^b^	Strong		Strong to medium		Strong
DC^c^	Strong		Medium		Medium
RWAP^d^	Residence practice is not relevant to whether the family has a male adult and the woman's distance from her parents' residence depends highly on her individual preference		Weak With limitations		Weak With relatively strong psychological support to natal parents but with time limitations

^*a*^PC, patrilineal-centered living arrangement; ^*b*^MC, matrilineal-centered living arrangement; ^*c*^DC, dual living arrangement; ^*d*^RWAP, residing without any parents.

The second dimension to measure this association relates to the range of families, which places further pressure on couples of the “one-child generation,” who may be required to provide care for all four of their older parents in the coming decades (Hu, 2019). Owing to the new “two- or three-child policy,” couples of the one-child generation may also face greater pressure due to care responsibilities for their children and grandchildren (Falkingham et al., 2020). Consequently, the extent of a family's expectations of their children's role in family caregiving is important because the latter must decide how to allocate their caregiving resources to both sets of older parents (Li et al., 2021). For example, suppose a family perceives their married daughter as part of her husband's family. In that case, the parents of the married daughter may lose significant family care resources in old age due to her absence (Guo et al., 2020). Therefore, this study considers “family expectations,” including the natal parents' views of their married daughter's role in the family, as a second dimension to analyze the interrelationship between living arrangements and family relations.

To measure how much family expectations contribute to the association between living arrangements and family relations, we include the following measurement criteria: (1) the woman's birth parents' perspectives of their married daughter's role in the family and (2) the surname of the children born to the woman (see Table 2). Here, our assumptions about strong family relations are identified when the woman is recognized as a family member by her birth parents and all her children inherit her family name. Intermediate family relations are identified if the woman continues to be regarded as a family member by her birth parents and/or if at least one of her children inherits her family name. Weak family relations are identified when the married woman is no longer considered a family member, and her children take her husband's family name.

**Table 2:** Basic information of the interviewees.

**Living arrangement**	**Number of people (%)**	**Have at least one brother**	**Live in the same village/neighborhood**	**Regarded as a natal family member**	**At least one child's surname born to mother**
PC^a^	53 (48.62%)	47	13/0	6	0
MC^b^	13 (11.93%)	0	13/13	13	12
DC^c^	25 (22.94%)	0	25/25	25	4
RWAP^d^	18 (16.51%)	11	0/0	12	0

^*a*^PC, patrilineal-centered living arrangement; ^*b*^MC, matrilineal-centered living arrangement; ^*c*^DC, dual living arrangement; ^*d*^RWAP, residing any parents.

The third dimension to explore the association between living arrangements and family relations refers to women's “ability to provide care.” As the population ages, family resources for caring for older people are being depleted due to the need to care for other family members and labor market demands. Therefore, from the women's perspective, their living arrangements affect their potential to care for older family members. Hence, deciding where or how to reside is important for reshaping their contributions as family caregivers (Lee and Luo, 2021; Xu, 2021). Studies on women's living arrangements have provided insights into intergenerational relations in Chinese families (He, 2019; Lee and Luo, 2021). However, a less detailed discussion exists on how living arrangements interact with women's family care contribution. Moreover, knowledge of the impact of dual living arrangements on intergenerational support is limited.

To measure how far family care contribution impacts the association between living arrangements and family relations, we examine a woman's ability to provide old-age care services to her natal parents (see Table 2). We assume that women's ability to provide old-age care services mediates their family relations with their natal family and justifies and negotiates their living arrangements. This study assumes a woman's ability to provide old-age care services greatly impacts family relations when a woman provides as much old-age care as possible to her natal family. An intermediate impact implies that the woman is highly likely to be able to provide old-age care services. Less impact is identified if the woman provides only temporary old-age care services or none.

### 3.2 Empirical strategy

Existing data were collated and reviewed by the research team of the Ethics Committee of Anhui Normal University (approval no. AHNU-ET2022048), using a template to ensure consistency. The source data included institutional regulatory documents such as the Report on Family Development in China (National Health and Family Planning Commission, 2015), the Population and Family Planning Law of the People's Republic of China (Standing Committee of the National People's Congress of China, 2002) policy documents on Anhui Provincial Family Planning Regulations (Standing Committee of Anhui Provincial People's Congress, 2002), and the Special Support Policy for Family Planning (Xiuning Health Committee, 2021).

In addition to institutional regulations, the empirical basis of this study included observational data from 109 semi-structured interviews of married women (aged 22–89 years), which were conducted in 2019 and 2023. We conducted a series of semi-structured interviews. Field interview focused on study's framework and with the four types of living arrangements of this paper. We arranged interview context based on interview topics, including (i) the prevalence of living arrangements for respondents and the impact on family relations from the women's perspective; (ii) specific information on how respondents access different living arrangements in rural areas; (iii) whether respondents receive support from their family in their living arrangements; (iv) the extent to which the local government promotes specific living arrangements if any; (v) the extent to which local communities encourage respondents to contribute to family care, and (vi) how far respondents themselves integrate living arrangements and family care contribution. We also asked open questions as how far respondents feel about their current living arrangement, how satisfied they are with such arrangement or any enthusiasm to change. A template was used for data collection to retain all data sources. Data sources included in the template were examined to assess, as far as possible, the internal consistency and any inconsistencies between different sources.

In-depth semi-structured interviews were conducted with married women (aged 22–89 years) in Huangshan in Anhui Province, South China. We used snowball sampling to select respondents (Robert, 1975). In Huangshan, officials from the Huangshan Civil Affairs Bureau were asked to recommend respondents that met the following criteria (1) living in the village for more than 3 years, with an average out-going time of no < 3 months per year; (2) engaged in at least one of our targeted living arrangements; (3) having contact with original family members in the last three years; and (4) willing to participate in this survey after understanding the purpose of the research. After women expressed their willingness to participate in our survey, the research assistant employed the semi-structured interview process. After completing the interview, the women received a random red envelope worth ~US $0.70 and US $2.80 as incentive. With the assistance of local officers and the local government's human resource departments, research assistants invited married women to participate in the survey from villages.

The interviewees' anonymous information is presented in Table 2. The interviewees comprised 109 women with various living arrangements. The Huangshan region was selected because population aging in this region is progressing at an intermediate pace compared with the rest of China. Its socioeconomic development is average compared with other Chinese regions (Huangshan Local Chronicle Compilation Committee, 2010). This region has a particularly long history of a strong Chinese traditional cultural impact on living arrangements, especially in rural areas. The author of this article has been studying the Huangshan region for more than 5 years and observing the living patterns of married people there for at least 2 months each year since 2019. Anhui Province was selected because it is one of the provinces where distinctive living arrangements have developed over time.

Interviewers were university students who had been well-trained in interviewing skills and had experience conducting resident interviews over 2 years. The research team selected and evaluated all the interview questions before being implemented in the interviews. The study protocol was approved by the Ethics Committee of the Anhui Normal University of China (approval no. AHNU-ET2022048) and conducted as per the ethical principles regarding human experimentation in the Declaration of Helsinki. All participants provided informed consent before study participation and could discontinue participation at any time for any reason. Each interview was conducted in person and lasted between 40 and 100 min.

The interviews were recorded for later transcription, and consent was obtained from all the interviewees before recording. Reports from each interview were prepared in Chinese, translated into English, and cross-checked by a professional language editing service. The reports from the interviews were thematically analyzed and initially coded for the following topics, paying attention to variations in how far living arrangements contribute to family relations via the mediating impact of family care contribution. The interview data were coded using a data analysis procedure, which included the preparation, training, and evaluation for coders, independent coding by trained coders, and verification and implementation of the coding results. All qualitative content analysis was done manually by authors.

- Decision-making process on living arrangements and changes in women's lives before and after marriage regarding family relations with their natal family.
- Women's natal parents' support for their new families and women's organization of family care service arrangements for their parents as well as plans for supporting aging parents.
- How far are women willing to move from their current residence to accommodate a new living arrangement? (open-ended question).
- What do women think about future living arrangement changes due to family care needs? (open-ended question).
- To what extent do women consider family expectations of their family roles and relationships after marriage? (open-ended question).

## 4 Empirical findings

### 4.1 Nexus of women's living arrangements and family relations

#### 4.1.1 Family structure

The interviews revealed that married women under patrilineal-centered living arrangements had at least one brother who proactively cared for their parents (Interview 2020-8; Interview 2022-16). These women left their natal family after marriage to live in the home of their husband's family, which may or may not have been in the same village as their natal family. Our analysis demonstrated that this living arrangement led to weak or intermediate family relations in the women's natal family (see Table 2). For example, an interviewee expressed the situation with her natal family after experiencing new living arrangements following marriage.

“I have visited my parents' apartment for only a short time during the New Year festival since I got married. I have to take care of my own family with two children and my parents-in-law, so I barely have time to think about other things. Sometimes, I make a phone call to my mother to ask if everything is fine. My mother has the traditional perception that she wants her son to take care of her, regardless of how often I visit her” (Interview 2020-1).

However, in the matrilineal-centered living arrangement, we found that for women who do not have brothers, their parents prefer to bring their husbands to reside with the women's natal family. Moreover, the women's parents offer new couples at least a room or possibly a home, which is usually in the same village. In addition, multiple interviewees commented that the new couples do not need to visit the husband's parents after getting married:

“My husband moved to my room after the wedding. My parents bought us some new furniture and articles for daily use, and we have hardly ever visited his parents since the wedding. His parents do not ask us to visit them since they also believe my husband belongs to my family after the wedding” (Interview 2020-18).

Therefore, based on the two indicators in this dimension, we propose that this kind of living arrangement leads to strong family relations from the women's perspective (see Table 2).

The dual living arrangement has appeared over the past decade and has been mostly adopted by daughter-only families. Since the families of both the woman and her husband prepare rooms for them, the woman can keep her old belongings with her natal family, and there is no need to move them to the new home. In principle, the new couple should alternate living with each set of parents, but most new couples in our study spent more time with the woman's parents. This is because the women were more comfortable and had a higher quality of life when living with their natal parents than with their parents-in-law (e.g., Interview 2019-4; Interview 2019-5). Moreover, they believed their natal parents could care for their children better (e.g., Interview 2019-1; Interview 2020-13), and their new home was often close to their parents' home. For example, Mrs. M said, “my mother-in-law and I have different food preferences. She likes soft food while I like spicy food, so do my children. I do not think I can bear the food she cooks…I have to say I am used to the food my mother cooks” (Interview 2019-14). However, this new living arrangement also led to family conflict.

“I have only one son, and my daughter-in-law has always lived with her natal family after marriage. I asked her to come back to our apartment, but she said, ‘I am the only child in my natal family, so it is natural to adopt a dual living arrangement.' How can this be! I prepared a new apartment for them and gave my daughter-in-law many valuable betrothal gifts. If she likes the dual living arrangement, I wouldn't give her so many things!” (Interview 2022-12).

This study demonstrated that the dual living arrangement strengthened women's relations with their natal families without an adult son and when the married daughter's new residence was close to her natal family's residence (see Table 2). However, this living arrangement differed from the matrilineal-centered living arrangement, as new couples were free to choose how far they resided from their birth parents based on their individual preferences. However, in reality, the dual living arrangement changed the structure of the husband's family. Older people, strongly influenced by traditional family ethics, believe that this living arrangement undermines the power of the husband's family because they lose their strict control over their daughter-in-law (Wong, 2016).

Women residing independently did not have a consistent natal family structure, and having a brother was irrelevant. Whether they had their own rooms in the natal family home depended on their preference. For example, Mrs. W had an elder sister and a younger brother. After she graduated, she found a job in the city and settled there because that was her preference. Her parents, who resided in a rural area, had kept the rooms of their three children intact, and she regarded this room as a temporary residence during the Chinese New Year (Interview 2023-1). Therefore, this study exhibited that such a living arrangement was irrelevant to whether the family had a male adult child. The distance between the women's residence and their parents' homes was highly flexible, as determined by their individual preferences (see Table 2).

#### 4.1.2 Family expectations

In the patrilineal-centered living arrangement, the forced change of living arrangement and the fact that the married women could not live with their natal family isolated them and reduced their opportunities for communication with their family. The married women lost possession of their room and belongings in their natal family home after marriage. For example, after Mrs. X married, her room became her nephew's bedroom, and her elder brother and sister-in-law took over the bed and bedding. Mrs. X said, “my mother pushes to me to leave their apartment right after dinner almost every time. I still remember that once I joked to say that I want to stay in her apartment overnight, but she seriously replied that she has no room for me.” (Interview 2019-7). Moreover, the local people in the village believed that allowing a married daughter to stay overnight in the family home brought misfortune to the family (Qing, 2014):

“I have often visited my parents' place over the past 20 years. However, I have hardly ever stayed overnight as I do not want to be blamed for bringing misfortune to the family, although I do want to stay there sometimes” (Interview 2019-8).

Regarding the surname of children born to a married woman under the patrilineal-centered living arrangement, the children did not inherit the mother's family name, and the married women forfeited the right to inherit property from their natal family. “I am married. It doesn't matter to me how my parents deal with their property. All things will be inherited by my brother eventually,” said Mrs. Q (Interview 2020-2). This situation meant that the woman's natal family no longer considered their married daughter a family member, weakening family relations from the women's perspective.

In the matrilineal-centered living arrangement, the daughter–parent relationship was strengthened through daily activities (as they resided together), and the parents recognized the married daughter as a family member. “My daughter stays with me after getting married; of course, she is our important family member,” said Mrs. S (Interview 2019-22). According to traditional norms, the grandchildren must take the wife's family name under matrilocal marriage, but accommodation can be made if the new couple has more than one child. However, the couple must discuss and agree on how the next generation will inherit the family names. For example, Mrs. Z had two daughters. The elder child followed Mrs. Z's family name, whereas the younger followed her husband's family name (Interview 2019-6). However, Mrs. Y and her husband agreed to allow their son to inherit the father's last name and their daughters to inherit the mother's last name. Having two daughters and one son, they could do as planned (most Chinese children do not have a middle name; Interview 2019-11). It was observed that the interviewees perceived a sense of continuity in their family lineage when their children assumed their parents' family name. This point still had a strong appeal to them, and the inheritance of the family name made the older generation feel a stronger intergenerational connection. In short, this study demonstrated a relatively intermediate to strong relationship between living arrangements and family relations in this dimension (see Table 2).

The woman living in a dual living arrangement believed that the relationship between a daughter and her parents would not change because of the daughter's marriage; the daughter was always a member of the family, and so was her child (Interview 2021-15; Interview 2022-3). As the patrilocal lineage relationship was considered replaced by the blood relationship of the women who chose this type of living arrangement, their families usually did not seriously consider the family name of the successive generation because the children shared their blood anyway. Consequently, most of the children of young couples took their father's last name. “My daughter's child is my grandson. I see him grown up, and we have a very close relationship. I do not care about his last name; he is just my grandson, that's it,” said Mrs. C (Interview 2020-14; see Table 2). In our interviews, several wives' families expressed that they voluntarily allowed their children to bear their father's last name to promote a traditional Chinese cultural background ideal within the new family. “I refused my daughter's suggestion that her second child inherit our family name. This does not matter to me. I want her two children have the same family name just in case the kids feel queered when having different family names,” said Mrs. F (Interview 2021-20). Besides, based on the traditional Chinese concept of family surnames, children inheriting their mother's family name means that the father is a Shangmen Nvxv, which is a stigma against the father's family. While the traditional emphasis on the heritage of children's family name has weakened, the married woman's family usually makes concessions in this regard, considering the emotional pressure on the husband and the stability of his family.

When couples resided without any parents, self-dependence was frequently pursued among married women, who played the role of the daughters of their birth parents instead of as family members. The women in this living arrangement were also unconcerned about the family names of their children. Although children born into this living arrangement follow the social conventions to take the husband's family name, one of them would accept the woman's family name in some cases. For example, as Mrs. B expressed, “my sister has only child who inherited her husband's family name, but I have two, and I gave one of my children my father's family name” (Interview 2020-19). Nevertheless, the findings of this study exhibited relatively weak but flexible family relations in this living arrangement, which also depended on how the women's birth parents considered their married daughter's role in the family after marriage (see Table 2).

#### 4.1.3 Ability to provide care

In the patrilineal-centered living arrangement, the women contributed less to their natal parents since the adult son's family was expected to take full responsibility for parental care, while the married daughter was more like a member of her husband's natal family. For example, Mrs. L hardly offered old-age care services to her birth parents because, as she said, “my brother is responsible for checking our parents' health condition and ensuring they have accessible medical care services when necessary. I have moved out after marriage and live within a nuclear family far from my parents' apartment. My job is to visit them during the festival, such as our New Spring Festival” (Interview 2020-17). Of course, Mrs. L also offered emotional support when needed owing to filial piety, as she said, “I often call to talk to my parents on the phone and check on them online. I called almost every afternoon during the days they were in a bad health condition.” The patrilineal-centered living arrangement was observed to weaken both the family care contribution for the woman's natal family after marriage and the mediating impact on the relationship between the woman's patrilineal-centered living arrangement and family relations with her natal parents (see Table 2).

In the matrilineal-centered living arrangement, young couples who resided with the wife's parents provided them with old-age care services until death. Meanwhile, the husband's parents provided no support to the young couple, and the couple was not deemed responsible for providing old-age care to the husband's parents. The husband was regarded as a Shangmen Nvxv after marriage, who is expected to contribute more to the woman's family. The family care contribution was concentrated not only on the husbands who integrated into their wife's families but also on young couples. The women in such kinds of marriages and living arrangements also had a greater family care contribution since they believed that this reflected filial piety and parental obligation. For example, Mrs. Z and her husband shouldered the responsibility of caring for her parents, including paying their pension contributions and daily expenses and sharing the housework (Interview 2019-6). In short, the matrilineal-centered living arrangement was observed to strengthen the women's family care contribution (see Table 2). Therefore, the women's relationships with their natal families were strongly enhanced by their family care contribution to this living arrangement. The following excerpt supports this finding:

“It is my responsibility alone to take care of my parents since I am the one who remained in my parents' family. I married my husband and took him to my family since I do not have a brother. However, instead of asking my parents-in-law, whom I have not visited for a long time, I ask my sister for help to care for my children when I am too busy and when my parents are sick” (Interview 2020-21).

The dual living arrangement generally allowed the young couples and parents to have flexibility in caring for each other. In principle, couples can provide old-age care services to both sets of parents. However, in this study, after 5 years of observation, it was evident that the women under the dual living arrangement preferred to spend more time with their birth parents. Consequently, those who spent more time with their natal family developed a better family relationship and took more time to care for their parents. For example, one interviewee stated,

“I must support my parents when they become old. Both my sister and I are willing to provide old-age care services to them, but I will provide more since I am the elder sister. I prefer living with my parents since I feel more relaxed here; we paid for their medical costs when they had surgeries, and I do household chores for them sometimes. I took care of dad for seven years until the end” (Interview 2020-12).

The relations between the women and their parents were strengthened when providing family care services. The women believed that they lived a more comfortable life in their birth home than in the home of their parents-in-law because the women and most family members on the husband's side regarded married women as brought to the husband's family. Therefore, married women are expected to contribute to the husband's family based on traditional cultural ideals. This explains why married women act more carefully in their husbands' families while feeling comfortable and relaxed in their natal family. Additionally, social stigma is relevant to married women's care contribution to the husband's family, as neighbors and relatives might judge women's filial piety based on this contribution. Nevertheless, the young couples also spent time with the other set of parents and assumed responsibility for old-age care when necessary (see Table 2).

Notably, this study revealed that most of the young couples who participated in the labor market resided without any parents and concentrated more on their careers (Interview 2020-3; Interview 2021-6). They maintained a certain distance between their own home and that of their birth parents, visited both sets of parents only during holidays, and spent less time taking care of them unless health emergencies arose. Couples residing without parents had a higher income and educational level than the other couples and considered having a work/life balance more important. In our interviews, some women in such living arrangements tended to use market services to care for children and older people. In this case, the traditional obligation of caring for one's family was psychologically translated into financial support and performed as an emotional expression between family members.

For example, Mrs. J's mother broke her leg 6 months ago. Mrs. J, who lived in a city different from where her mother was, and her sister, who lived overseas, alternated caring for their mother at her home for 1 month. However, Mrs. J later returned to work and only spoke to her mother by phone over the subsequent months. She hired her mother's neighbor to care for her mother. Hence, the care she provided to her mother focused on psychological comfort (i.e., comforting her and talking to her regularly) instead of hands-on care services (Interview 2019-9). In summary, this study observed a weaker family care contribution when the couples resided without any parents but had relatively strong psychological support, representing an important change in care support for the natal families of married women (see Table 2).

Women's ability to provide care for older family members is significant in an aging society. The premise is that the relatively weak state and backup community care services are not priority choices when individuals have care needs under traditional cultural ideals. A prevalent cultural value suggests that older couples may be placed in institutional care or community care due to their adult children's lack of filial piety, as they fail to fulfill their obligations to take care of older relatives. Nevertheless, this study observed that women's ability to provide care improved family relations by increasing their contact with older family members and engaging in emotional exchanges with them owing to their living arrangements. The more frequent the care contribution by the women, the higher the willingness of older people to take care of their grandchildren. Older people in rural China increasingly rely on family care and try to improve family relationships with the younger generation since they may spend less time with their adult children who migrate to cities for career opportunities (Chen et al., 2011). The married women tended to choose living arrangements closer to their parents to increase their family care contribution. This means that their ability to provide care mediated the association between the women's living arrangements and family relationships with their natal parents.

### 4.2 How far living arrangement interact with family relations

The interviews revealed that, first, in rural China, the family structure is an important prerequisite for family relations between married women and their birth parents. In patrilocal marriage, married women, when they have biological brothers in the natal family, are often forced to cut ties with their natal family and integrate with their husband's family soon after marriage. Traditional cultural ideals inhibit married women from maintaining close relationships with their natal family; otherwise, they may be suspected of trying to gain inheritance property from their biological brother, which might lead to a lack of family harmony (Fei, 2010). Besides, married women are expected to spend most of their time and energy on their husband's family. Nevertheless, owing to low fertility, especially for the one-child generation, the husband's family finds it hard to keep this traditional cultural ideal. Therefore, the daughter-only family could maintain a close relationship with their married daughters.

Second, the proximity of living arrangements is a powerful predictor of family relations. Women who provide significant family care to their parents, or vice versa, usually reside with their birth parents. Compared with women who reside close to their parents after marriage, the differences in the living environment and lifestyle of women who reside in independent homes and their parents' homes were significant (Li, 2003; Du, 2021). However, they all provide family care and support for their parents in different ways. While residing without any parents does little to enhance family relationships, it contributes to the gender harmony of Chinese married women since they have more flexibility and freedom to choose their role in family relations.

Third, in terms of family expectations, breaking the expected societal rules for married women improves the family and parent-child relationships, which contradicts the belief that family expectations promote intergenerational relations (Roberts et al., 1991). For example, matrilineal-centered and dual living arrangements allow both the married woman and her children to inherit the home and assets from her natal family. Therefore, the family breaks traditional family expectations by empowering their daughter to act as a male adult family member, strengthening the relationship between the daughter and her natal family. However, the strength of family relations differs between matrilineal-centered and dual living arrangements. The former promotes the married daughter's family status by forcing her to play the “son's” role in the family, which means a change in the daughter's social attributes, and by forcing her husband's family to accept that the husband will become part of the wife's family forever. By contrast, the latter empowers the daughter with family rights by accepting that a married daughter can play the same role as a son in the natal family. The husband is free to decide his level of involvement in the wife's natal family. Therefore, a young couple's individual preferences largely shape the strength of family relations in dual living arrangements. This study observed that the matrilineal-centered living arrangement is the best arrangement to strengthen the relationship between married women and their natal families. However, most rural families choose to practice dual living arrangements. Thus, the cultural ideal of marriage impacts people's choices markedly and they adopt a dual living arrangement to eliminate the concerns of the husband and his family and strike a balance. This choice of living arrangement has developed from the improvement in married women's rights and family relations. While the cultural ideal of filial piety is deeply integrated within Chinese people, women are trying to express it in a new way.

Fourth, a long-lasting and high-frequency family care contribution to birth parents ensures long-lasting and stable family care arrangements for women. This process brings no short-term economic benefits; instead, it costs women time, energy, and sometimes even money when they must give up profitable jobs to care for older people. According to the interviews, parents usually seek emotional support besides financial support, and high emotional support leads to strong parent-child relationships. Older people can turn to emotional and family pursuits when basic economic needs and aging support are met (Yan, 2003). In traditional Chinese culture, daughters are the best candidates for offering emotional support to the natal family, which is coherent with parents' needs in current society.

In addition, most of the interviewees belong to the “sandwich generation” whose current care needs are changing, as is their willingness to abide by the living arrangements and gender harmony ideals that were decided upon when they got married. We argue that women have a high potential to change their living arrangements within the four living arrangements discussed here. As revealed by the interviews, unmet old-age care needs and filial piety are other important factors that married women must consider when deciding to change their living arrangements (e.g., Interview 2019-13; Interview 2021-20). Therefore, more women of the “sandwich generation” in rural China may decide to live close to their birth parents to balance unmet family care needs, thus affecting family relations. Furthermore, women's family care contribution mediates their living arrangement decisions and family relations; consequently, their satisfaction of filial piety through family relations impacts their psychological judgment of being equally regarded as the female gender.

## 5 Conclusion

The present study focused on how women's living arrangements after marriage affect family relations in rural China. Previous research has found that most married women in rural areas change their status and become members of their husbands' families according to norms of traditional Chinese patriarchal society. They then contribute significantly to their husband's family relationships in patrilocal arrangements. However, the findings of this study indicated that in contemporary China, women greatly promoted family relations in their natal families when they practiced a dual living arrangement, and generally fostered family relations when under a matrilineal-centered living arrangement. Families that challenged the traditional social expectations of women tended to develop stronger family relations.

The interview data confirmed that women's family relationships could be improved further as they strengthen their ability to provide family care, experience different family structures, and face various family expectations. However, this kind of logic was not always practiced. In cases where women prioritize psychological support, while paid care services meet the care needs of older people, their relationships with their natal parents could also be strengthened. This situation has recently emerged in rural China, where women, on average, have higher incomes than in the last few decades. The concept of gender harmony in traditional culture has always influenced the life practices of rural women. However, significant changes have emerged under the influence of new family care demands. The concept of gender could be formulated, rejected, rehabilitated, and downplayed in the social formulation at different times in history, depending on the family structure, family expectations, and ability to provide care.

The family dynamics of Chinese women's natal families will evolve along their own trajectory, influenced by living arrangements and the need to navigate domestic challenges stemming from the family's geographical spread and regional deviations as the economy grows. The analysis and discussion in the present study demonstrate useful insights into both the role of women in maintaining family relations and the newer forms of living arrangements that influence this phenomenon. These insights are relevant to scholars and practitioners interested in women's rights, particularly those of rural women. This study proposes that the family care contribution plays a significant role in mediating the relationship between women's living arrangements and family relations. Furthermore, this can inspire the development of family living arrangements with Confucian cultural ideals in an aging society, in which the changes in living arrangements impact gender harmony as constructed under Confucianism in rural China. In particular, women under dual living arrangements, involving their as well as their husband's parents, contribute to avoiding conflict associated with gender-based problems in the family while balancing gender responsibility.

## 6 Limitations

As with other studies, this study also has limitations. First, it focuses only on the women's perspective; future research should include more groups of interview respondents, such as older parents and parents-in-law. More husbands should also be included when examining the study's research questions. Second, this study only includes interview data in its empirical analysis; future research should consider a broader database and include quantitative data by administering a questionnaire survey. Third, future research should also include both cross-sectional and longitudinal surveys to explore the short-term and long-term living arrangement development of Chinese couples and their relevant social acceptability conditions.

## Data Availability

The original contributions presented in the study are included in the article/supplementary material, further inquiries can be directed to the corresponding author.
